# The Physiological Therapeutics of the Borderland of Insanity

**Published:** 1900-12

**Authors:** C. A. F. Lindorme

**Affiliations:** Chicago, Ill.


### THE PHYSIOLOGICAL THERAPEUTICS OF THE BORDERLAND OF INSANITY.

BY C. A. F. LINDORME, PH.D., M.D.,

CHICAGO, ILL.

If we claim for the borderland of insanity a by far more extensive territory than is usually allotted to it, we believe that the related circumstances give us good ground in doing so. In Dr. Cullerre's book, "Les Frontières de la Folie," the outlook is not so vast. But the French *confrère* keeps on the thither side of the fence, treating the slighter cases of pronounced insanity. What we consider more important and also by far more interesting, is, to explore the hither range of the fence, the haunt of what I am inclined to call the *amateur-insane*, a fellow who has all the rough-hewn material of sensibility at his disposal, but for some reason or other chooses to give way on select occasions to the itch of firking mad. At any rate, it is the proper field to ventilate physiological therapeutics on, where the germ of the pathology is started, of which most likely within short some successful microscopist on the other side will discover the insanity-microbe of.

Jacobi (Siegen) was an early advocate of an extensive range of the borderland of insanity. A very amusing *quid pro quo* happened at his table with reference to this theory. Once he had as a guest a brother-superintendent, who was a very moderate man. When Jacobi wanted to fill his glass again he drew it back with the remark: "I have my eighth." On this utterance the whole table burst out laughing. The stranger was in a measure taken aback by the general hilarity on that score, and looked around inquiringly. "Pardon our laughter," said the house-physician, "but our worthy chief is of opinion that every person has his eighth of insanity, and when, by your remark, you so readily seemed to admit your share, we could not help referring it to the theory, instead of the wine. Hence our glee."

Nor is this matter any more at all *sub judice.* If Moreau de Tours stakes his authority as psychiater by the rash and uncanny assertion, that there is no such thing as three-quarter madness, full-face or profile, he cannot redeem it as scholar. Science has passed sentence on it; her emphatic denial of a strict line of demarkation between health and disease, by proving pathology only a deviation from physiology, extends likewise to the mental domain.

There is one point, however, as to which we must take issue with Jacobi (Siegen). The percentage of madness to which he declared everybody subject does not exhibit in any corresponding steadiness all through life, but is an average liability. It may show very differently, now, at par, or, for the matter of that, with a premium, now, in infinitesimal dose. Since the will is the point at issue, there is no telling how the shades will run, how close to full-fledged insanity, or how far from it.

The celebrated Italian crank-detective, Cesare Lombroso, bears a testimony which is rather fulsome. Changing his method and counting for shortness sake none but those sane in mind, it is among the great men none but the only Goethe who gets from him a clean bill of health, an outcome for which the German bard is little likely to be grateful to him. Goethe was not the kind of man to admire the negative virtue one is given credit for in the "good society" of which he says:

"Man nenn t sie die gute,
Weil sie zum kleinsten Gedicht keine Gelegenheit giebt,"

(It is called the good,
Because it gives no occasion for the most minimal poem), a standpoint which is shared by the great compatriot of Lombroso, Dante, who, in his transcendental picture, has a particular arrangement for that class: Since they had not done anything to deserve them the heaven, they ought to go to hell. But the inmates there would take offense at a set which escaped damnation only because they had not the grit to be bad. And this Dr. Cullerre indorses with a prosaical argument: If the fools ever die out, the world will stand stock-still, not from too much wisdom, but from an insufferable *ennui*.

Repeatedly we have had occasion to emphasize the truth that physiology bears more of the striking features of real science than the remaining branches of medical learning. Yet, in the history of the science of medicine it did not come first. The disease-aspect preceded the healthy exhibit. Youthful imagination and early exploration will always first be attracted by the gruesome. And correspondingly it is not infrequently by pathology that we are led to the physiologically most relevant points. Now, our most experienced psychiaters unanimously contend, that it is not in the intellect, but in the emotional part of the personality that the symptoms of lunacy come first to show. Unexceptionally the alienation of the mind takes its starting point from an exaggerated qualification of the own personality, supererogation or disparagement, as the case may be, but always a display which has a bearing upon an eccentricity of selfhood. When a hypochondriac indulges in a heap of diagnostic nonsense and pathological absurdity, this is not because he brought the own etiology to an extremely fine point, but because it is *his* health which is concerned, *his* individuality which is called into question. He does not even try to exhibit to the adversary any cogency of reason in his conclusions. And, a highly important point, he never exerts himself to learn, nor ever profits by his experience; he grafts one fallacy upon the failure of the preceding one, and believes ever the new erroneous discernation the "true thing it really is." So, in mania of persecution it is not any reality of a motive of fear which causes the suspicion, but all there is in it is what the liveliness of the imagination of the insane can puff it up to. The personality of the maniac would not be up to the high-water mark where his exaggerated self-feeling wants it, if he was not the fascinating object of romantic hunt and haunt, and his imagination runs riot with the eccentric stories, which, plausibly or forcibly, lend themselves to the fantastical construing of the self-made victim. Logic, in this regard, the maniac does not even care for, although he knows to use his intellect when he is interested to do so. Tuke reports of an inmate of a lunatic asylum, who made a terrible mess out of some hundreds of millions of pounds sterling he owned in his fancy, but calculated correctly to the last farthing the interest on a few sovereigns he had deposited in a savings bank. Does not beside the maniac of greatness who thinks himself the lord of creation meekly interrupt his creation-business to conform himself with the order of the day as directed by the interne?

Following up our subject from the point of agreement of our excellent psychiaters, the exaggerated self-feeling of the incipient lunatic, we are so much more warranted in finding ourselves on the right road, the less we are satisfied with the *psychose* theory of the rudimentary metaphysicians who take care among the psychiaters of the didactic part of the business, and the more we look out for physiological vouchers of the organic exploration. Since alienation takes its rising from the emotional matrix, it cannot be qualified by outward impressions, except in conformity with inner disposition, and this agrees with the anatomical substrata of such a process, on the one hand the encephalon, as recipient of the outward impressions, on the other the blood-disks by which in the capillaries are actuated the nerve-cells, the blood-disks exhibiting the concentrated will living in the blood-producing organs of the body, the mouth and stomach with glandular outfit, the duodenum with liver and pancreas, the jejunum and ileum, and the spleen and the marrow of the bones, where the opinion is held now that the red-blood corpuscles are manufactured, which enumeration is so much less exhaustive, as there is much reason to believe that the solar plexus not only but also the sacral plexus, may claim a great share in the mentioned process. At any rate this physiological anatomy reappears again in the first manifestation of personality in its self-consciousness on the hither, the healthy side, or that which is reputed to be so. There is in human life, one should think, nothing more determinate than the will of man, nor is there, according to the general belief, nothing man knows so much about as his *will*. Again, there is nothing in which the human will is so obviously involved as *love*. But in spite of all this fancied certainty we read in Shakespeare about the latter:

> "Love is a smoke raised with the fumes of sighs ;
> Being purged, a fire sparkling in lovers' eyes ;
> Being vexed, a sea nourish'd with lovers' tears ;
> What is it else ? A madness most discreet,
> A choking gall and a preserving sweet."

Worthy to be quoted alongside of Shakespeare, we will mention Heinrich Heine, who, in a few lines, gives the most impressive qualification of the passion which we have ever come across with :

> "Die Engel nennen es Himmelsfreud,
> Die Teufel nennen es Hoellenleid,
> Die Menschen die nennen es Liebe.
> (The angels call it heaven's delight,
> The devils call it hellish plight,
> We men, we call it, 'be in love.'")

A never missing characteristic of the higher psychological states, those furthest apart from the brutish tendencies, is a lack of sameness, leaving man without a definite selfhood, splitting him up, as it were, psychologically, an amazement of his own thought, the thought about himself.

> "Love ! His affections do not that way tend ;
> Nor what he spake, though it lack'd form a little,
> Was not like madness. There's something in his soul
> O'er which his melancholy sits on brood."
>
> —*Shakespeare.*

Now, this enunciation of Polonius is all good and well so far as it goes ; it is practically clear enough ; it is to all intent and purpose a satisfactory symbolization of the condition of our mind referred to. Psychological empiricism can operate with it without diffidence and misunderstanding. But a theoretical argumentation cannot be built up on the defective disquisition. Here we must learn which is which, whether the soul is outside the melancholy, or the melancholy outside the soul.

One would suppose that there was nothing more intimate to man than man himself. But no sooner man has a psychological entanglement of an emotional kind, he gets lost in the question of his personality.

> "Tut, I have lost myself; I am not here;
> This is not Romeo, he is some other where."
>
> —*Shakespeare.*

And man is eager to avail himself of this uncertainty of unitary exhibition to shirk a responsibility which is bothersome.

> "Was't Hamlet wrong'd Laertes? Never Hamlet;
> If Hamlet from himself be ta'en away,
> And when he's not himself does wrong Laertes,
> Then Hamlet does it not, Hamlet denies it."
>
> —*Shakespeare.*

And it is a question whether moralizers have been just in blaming man for the lack of steadiness which his nature displays as an inborn feature:

> "Quel animal est l'homme! Il va du blanc au noir;
> Ses sentiments du matin il les condamne au soir"
> (What an animal is man! he shifts from blue to red;
> His thoughts on getting up, he spurns on going to bed.)

As usual, it is at the door of old metaphysics that the fault must be laid. It is owing to its lack of detail-information of the human nature. If we examine the vouchers of physiological anatomy, we find conditions which in no way militate against the purity of man's design. But the matrix of his evolution is differential; it is *constitutionally conflicting.* Since physiology teaches us that neither a nerve nor a muscle-actuation is found, or, indeed, possible, except through means of a blood-oxygenation, it follows that the individual exhibit must be according to the blood-supply, and this leaves room for ever so many inner and outward modifications which preclude all setting down man as a definite thing, like a clock which runs down as it is wound up. In society there were two factors who connived historically in this matter; transcendentalism and legislation joined hands to establish the pragmatism which, without unmitigated responsibility and merciless persecution by rote, could not be realized, and settled in public opinion an identity of individuality in fictitious continuity all through life, the truthlessness of which appears on every page of physiology and anatomy. The human individual is not a fixed, morphologically definite proportion, but A GROWTH, and must result according to the conditions of that growth. Again, the character or the personality of man is not something originally given, ready-made, a gratis-gift of nature, for the whole lifetime, but *something man must fight for;* it is *a mental achievement*, the primitive conditions for which are as often deteriorated as improved, and which cannot be improved except in keeping with the spontaneous efforts of the individual; the higher the rank, physiologically, of the individual development, the greater the stake of the personality, psychologically, in the contest. It was a man like Goethe in whom was loudest the wail of the mental conflict,

> " Zwei Seelen wohnen, ach, in meiner Brust." —*Faust.*
> (Two souls are, alas, lodged in my breast.)

And no less a man than Shakespeare left in his Hamlet the huge problem unsolved of the psychological order of thought and action.

There is in the distinction of man from the animal by the ownership of an endowment beyond the organs of sense, which we share with the animal, a drawback, as it were, from the very perfection which it represents. As a fact, the preferment of man by nature, the higher plane which is exhibited by his capacity of thought, the establishment in language-expression of a reflex reality, a fictitious world of abstraction, by a mistaking of the same for the world of reality of our surroundings, tends more to increase craziness than anything in human life, so that we must declare the very advantage of the man over the animal, an intelligence reaching beyond the organs of sense—an irony upon these—manifests itself in an organ of nonsense, which animals have not.

There has been in latter years a good deal of loose talk about the language of animals, and eager investigators have started on an exploration. Ants, bees, have, by preference, been selected for objects of observation. But we are inclined to believe that the explorers lack the principal condition of success in experimenting. This is the guiding insight into the material found. The said field is far from being without exploration. It is a generally admitted position that there is a communication between animals. But it is only a sympathetic soliloquy of sensation, not a communication of thought. The formation of thought which would have to precede does not take place. The animal language is that of gesture, precisely that which we have, too, and which we avail ourselves of in emotional states, the nature of which carries us beyond the applicability of language. Supposing a hen falls in with a particularly good spot for scratching. Now, she will utter sounds which are expressive of the fortunate discovery. The little flock will take it that way, too. But yet there was not a communication of thought; it was simply an utterance of joy, such as the small ones might have uttered themselves as the expression of satisfaction over the fortunate chance. This agreement of sensation constituted the basis of understanding. Similarly in other cases. Supposing the old hen had seen the hawk. She would incontinently have uttered the very cry of warning which would have collected the little ones under her wings. But yet it would not properly have been a call. The hen herself, by the sight of the hawk, would have been frightened, and the ensuing utterance worked as a warning, because the little ones, by a similar impression, would have been prompted to a similar utterance; hence, their understanding and their running for protection.

The point at issue in the language of an animal is always an emotion. The communication does not refer to a generally expressed opinion, but to a specially experienced desire, and the like holds with small children, deaf and dumb, &c. Thus, bringing it down to a fixed rule, in defining scholasticism, we have to say that, if animals have a language, and seem eventually to avail themselves of it like man, it is nevertheless always language without grammar, and this is the point of distinction we have to note as the characteristic one. Nor do we consider it a truism to emphasize the appallingly small number in every country of highly gifted and exceptionally learned scholars who understand fully their own language, so that a more specifically distinguishing mark of the human language is to the utmost degree indicated, and such a mark we find in the rule, that *the word of human language is a classification.* The thought conveyed by language can never be well understood, but creates confusion, if we fail to have regard to this point. By most people it is ignored. Grammars even, do not distinguish rightly. They distinguish abstract nouns from other nouns, while really there are no other but abstract nouns; the circumstance that all words of the language are a classification, or refer to such a one, stamp them abstractum. One exception only has to be stated. This is the proper noun—the name. But this is an exception which confirms the rule, because it is in a language an extra-systematic accessory; it is not subject to translation, and is no thought. It is a label.

Considering the importance of this matter, opposite the lack of plainness, among the learned, even, it will be useful to give an an example. Supposing somebody would not know what is William McKinley. Well, the most thorough mode of information would be to buy a ticket to Washington, D. C., go to the President, and tell him, your honor, there is a man who does not know what is William McKinley. Allow me to make him acquainted with you as the individual in question. "All right," says Mr. McKinley, "you are perfectly welcome, but be a little quick about it, because my public duties lie in a bit different line."

Now, this illustration, by exhibiting the President of the United States, would have given in full the explanation of the noun, or the proper name, William McKinley. The latter would not have been exhibited as a specimen of some kind, but the congruent identity itself of the word. To go to Pittsburg would not have served the same purpose as going to Washington.

Now, supposing on the other hand, that a person does not know what is a chair. Would it be sufficient to show him the first one at hand to give him the congruent identity of the contents of the word? By no means. It would not be sufficient, even, to take him to a furniture store and show him all the makes there; or to an exhibit of all the varieties in use, because the word "chair" comprehends also the chairs which are not made any more; nay, the chairs, even, which may be later invented or come in use, and this goes far to show that the word chair means no special, individual chair, but is, as we said, a classification. It means a chair is a sort of a seat, and to go for "the chair" is an absurdity; there are concrete exemplifying chairs enough; but "the chair" (as such) remains forever an abstractum. But this scientific order and methodological proviso is often not apprehended by men, even, who consider themselves scholars of purest water. There are men, who, satchel in hand, go for "matter." Seriously they set about finding it, and are tormented by a sort of compunction that they cannot realize for the progress of science a successful scraping up in some obscure corner of a sufficient quantity to prove its existence, and a long wail goes through the most learned literature that it is impossible to find it. Now, if a definition of matter is demanded we can give it in the words, that it is the most general mode of form-perception, or the perceiving of bodily quality; matter is the symbol for the universal class of material things, by which comprehensively a certain condition of all of them is understood, and now comes a discontented inquirer, and wants to find the symbol again as a special thing. This reminds me of an anecdote which the students tell of Hegel, the vocabulating professor of the old metaphysics, the rudiments of which hamper yet all honest and earnest philosophical work, and adulterate scientific progress. Coming to a fruit store, Hegel says:

"I want some fruit."

"All right, sir, we have apples, pears, very nice grapes, plums, apricots, all that is in season."

"I do not want apples, pears, grapes, apricots, plums; I want fruit, fruit, fruit, *fruit as such.*"

The 500 to 600 students Hegel had at every lecture were stunned at this kind of language. The apple-stand man set the professor down as a fool:

"Was der Verstand der Verstaend'gen nicht sieht,
Das uebet in Einfalt ein kindlich Gemueth." (Schiller).
(What the intelligence of the learned not knows,
A kid acts thoughtless with the skill of its toes.)

And the wise, that is the man who wants to learn, can find in the works of the last quoted admirable bard a wonderful distich which by itself is sufficient to satisfy the most critical of philosophical investigators of matter, as distinguished from mentality:

> "Leicht bei einander wohnen die Gedanken,
> Doch hart im Raume stoszen sich die Sachen."
>
> (With ease our thoughts do locate by another,
> But hard in space does one thing hit the other.)

There is no field of pathology so denuded from prophylaxis as mental disease. Nay, worse than that; there is not only no prophylaxis in mental disease, but the borderland of insanity, in its *amateur-insane,* foster a class of people who use considerable painstaking in preparing the soil for the regular lunatic patient. Hence the necessity to bring physiology to bear upon the matter. The wisdom of the old rule holds here more than anywhere, *principiis obsta,* or, in plain English version, "one stitch in time saves nine."

In medicine, since the time when the uncouth word, ABRACA-DABRA, written on the recipes of medicine by the gallon, or hung, as amulet, round the sick, was considered the most reliable safeguard against the devil, who, at that time, for the romanticism in medicine, did about what does the deadly microbe now, a good deal of purification of language has been accomplished. A counterpart of the funny lament of certain Hegelian epigones, that matter cannot be found, was exhibited in the vitality-theory. But the adherents to it insisted on the concrete existence of this symbolization. An explanation is easily found in the circumstance that either word is a classification. This the vitalists ignored. The matter-desperate forget it. That which exists in concrete is the groups of the conditions which have been symbolized by the words. Vitality exists, the therapist knows, that it is something he can count within the young, and that it is something which fails him in the old. But vitality as such is an abstractum, and it is Don Quixotic to send to the drug store for fifty cents worth of vitality. Matter exists too. There is nothing in nature more easily found than it. But a shop which keeps matter on hand for the drowsy out-of-time Knickerbockers whose abstraction-jangle has a wrong tune and a false key, there is none, and being confronted with a case that wants correction, one is inclined to regret the reformation of Pinel which brought it out of use to manage such correction by an *argumentum ad hominem* in the shape of birch in most personal application. To despair in this world of finding matter is just as wise as to walk among the trees and then clamor for the wood; on the face of the earth it is as impossible to find an acre of wood as such, as it is impossible to feed on fruit as such. It is as impossible as to find the soul or spirit as such, all the length of time notwithstanding that our anatomical forerunners have been at it to detect the soul in the pineal gland, and to dissect it out from it, and hand it round the boys in the amphitheatres. The soul can be taken out of a man, and the spirit out of a horse, by treating the man mean, and running the horse down. But then the body goes too, in either case. But be the condition of things changed as it may, a separation of the soul, or spirit, from the body, is there as little as the force can be taken from an ox, so that the force is there and the ox in an adjoining stable, or the hunger from a baby, so that the baby is here and the hunger in a bottle alongside. Whether we speak of the sweetness of the voice of a women, of a lark, or a nightingale, or of diphtheria, it is a symbolizing of groups of conditions, and to deduce from such symbolization in vocabulating abstraction a corresponding identity of concrete special existence in outward reality is methodologically a silliness, just *amateur-insane.*

From the nature of the world it can easily be concluded that language cannot be used intelligently for sensible communication, unless with every word the same conditions are presupposed which were originally understood, or, if a change takes place, that it be a reciprocal or mutual one. In all important discussions particular care should therefore be had to avoid such eventuality by referring all symbolization back to the comprehended conditions.

I must conclude. The editor has reason to chide me for my lengthy article. But I crave permission to submit one point more. The proposition above implies that man is also a group of conditions, and this issue would hurl the problem of a unity of physiological propositions and psychological emotions, of nature and civilization, back to the fathers from whom we inherited it as the quandary of social evolution: In the last century the last word which was said in the matter was, *L'Homme machine*. Nor has this century so far brought the release. The occasion for this in matter-of-fact philosophy is however by no means out of the question. If man, as a part of nature, seems subjected to her ways, yet there is a sweeping difference: There is no machine, nor can any one be made, which, like the machine represented by man, possesses the *capability of self-culture.* And this difference establishes the point of distinction. The very fact of this ownership imposes upon man a duty, *the duty of self-culture,* and from this follows his *responsibility anent society.*

This is the point of the new departure of law and social ethics.

